# The rumen microbial metagenome associated with high methane production in cattle

**DOI:** 10.1186/s12864-015-2032-0

**Published:** 2015-10-23

**Authors:** R. John Wallace, John A. Rooke, Nest McKain, Carol-Anne Duthie, Jimmy J. Hyslop, David W. Ross, Anthony Waterhouse, Mick Watson, Rainer Roehe

**Affiliations:** Rowett Institute of Nutrition and Health, University of Aberdeen, Bucksburn, Aberdeen, AB21 9SB UK; SRUC, West Mains Road, Edinburgh, EH9 3JG UK; Edinburgh Genomics, The Roslin Institute and R(D)SVS, University of Edinburgh, Easter Bush, Edinburgh, EH25 9RG UK

**Keywords:** Archaea, Illumina, Metagenomics, Rumen, Succinovibrionaceae

## Abstract

**Background:**

Methane represents 16 % of total anthropogenic greenhouse gas emissions. It has been estimated that ruminant livestock produce ca. 29 % of this methane. As individual animals produce consistently different quantities of methane, understanding the basis for these differences may lead to new opportunities for mitigating ruminal methane emissions. Metagenomics is a powerful new tool for understanding the composition and function of complex microbial communities. Here we have applied metagenomics to the rumen microbial community to identify differences in the microbiota and metagenome that lead to high- and low-methane-emitting cattle phenotypes.

**Methods:**

Four pairs of beef cattle were selected for extreme high and low methane emissions from 72 animals, matched for breed (Aberdeen-Angus or Limousin cross) and diet (high or medium concentrate). Community analysis was carried out by qPCR of 16S and 18S rRNA genes and by alignment of Illumina HiSeq reads to the GREENGENES database. Total genomic reads were aligned to the KEGG genes databasefor functional analysis.

**Results:**

Deep sequencing produced on average 11.3 Gb per sample. 16S rRNA gene abundances indicated that archaea, predominantly *Methanobrevibacter*, were 2.5× more numerous (*P* = 0.026) in high emitters, whereas among bacteria Proteobacteria, predominantly Succinivibrionaceae, were 4-fold less abundant (2.7 *vs.* 11.2 %; *P* = 0.002). KEGG analysis revealed that archaeal genes leading directly or indirectly to methane production were 2.7-fold more abundant in high emitters. Genes less abundant in high emitters included acetate kinase, electron transport complex proteins RnfC and RnfD and glucose-6-phosphate isomerase. Sequence data were assembled de novo and over 1.5 million proteins were annotated on the subsequent metagenome scaffolds. Less than half of the predicted genes matched matched a domain within Pfam. Amongst 2774 identified proteins of the 20 KEGG orthologues that correlated with methane emissions, only 16 showed 100 % identity with a publicly available protein sequence.

**Conclusions:**

The abundance of archaeal genes in ruminal digesta correlated strongly with differing methane emissions from individual animals, a finding useful for genetic screening purposes. Lower emissions were accompanied by higher Succinovibrionaceae abundance and changes in acetate and hydrogen production leading to less methanogenesis, as similarly postulated for Australian macropods. Large numbers of predicted protein sequences differed between high- and low-methane-emitting cattle. Ninety-nine percent were unknown, indicating a fertile area for future exploitation.

**Electronic supplementary material:**

The online version of this article (doi:10.1186/s12864-015-2032-0) contains supplementary material, which is available to authorized users.

## Background

Methane is a greenhouse gas (GHG) with a global warming potential 28-fold that of carbon dioxide [1]. It is responsible for 16 % of total anthropogenic greenhouse gas emissions [2]. Ruminants are the major producers of methane emissions from anthropogenic activities, accounting for 37 % of total GHG from agriculture in the UK [3]. Lowering methane emissions has therefore become a major priority in ruminant livestock production, with many different strategies having been proposed to mitigate emissions, including different dietary formulations, chemical and biological feed additives, chemogenomics and antimethane vaccines [4–6]. Research is also under way to determine the extent to which the animal itself has control over its ruminal microbiota, with the intention that, if the trait is heritable, low-methane livestock phenotypes may form the basis of a breeding programme to produce ruminants with a smaller environmental footprint [7]. If any of the strategies proves to be successful, benefits may be anticipated in energy retention by the animal [3, 8].

Methane, although considered to be an atmospheric pollutant, is a natural product of anaerobic microbial fermentation [9]. The rumen is an anaerobic microbial ecosystem in which a dense mixture of protozoa, bacteria and anaerobic fungi convert carbohydrates to short-chain, volatile fatty acids (VFA), which are absorbed by the animal and used in energy metabolism and protein synthesis. Hydrogen is formed as a result of fermentation, and it is used by methanogenic archaea to reduce CO_2_ to methane [10]. Hydrogenotrophic methane production is quantitatively the most important source of methane, although methylotrophic methanogenesis also occurs, forming methane from molecules like methylamine [11].

Deep sequencing of DNA extracted from complex microbial communities enables many aspects of microbial ecology to be determined. Metagenomics allows the abundance of all genes present in the microbial community to be determined and metabolic pathways to be predicted. The first reports of metagenomic analysis of ruminal digesta demonstrated the power of the technology, focussing in functional terms on fibrolytic enzymes [12–14]. Since then, several further reports have appeared, applying various metagenomics methods again to fibrolytic enzymes [15–17], and to lipases [18, 19], virulence and antibiotic resistance genes [20], polyphenol oxidase [21], CRISPR elements [22] and secretome proteins [23].

In the present paper, we apply metagenomics to analyse the root causes of different methane emissions in high- and low-emitting beef cattle. A recent metagenomic analysis of high- and low-emitting sheep found correlations between the metatranscriptome rather than gene abundance and methane emissions in sheep [24], contrary to the expectation that methanogenic activity should be proportional to the abundance of methanogenic archaea and/or H_2_-producing microorganisms [25]. Several other studies have failed to find such a relationship [6, 26–28]. The present study was undertaken to investigate the comparative metagenomics of high- and low-emitting beef steers. The animals were selected as pairs of the highest and lowest emitters from an experimental group comprising two cattle breeds each receiving one of two diets differing in concentrate content. Hydrogen and methane emissions from the whole group were reported previously [29], as was a preliminary correlation between methane emissions and the ratio of total ruminal archaea to total bacteria [30]. Unlike the other studies, archaeal gene abundances in ruminal digesta, including 16S rRNA genes, corresponded with the extreme differences in methane emissions from beef cattle. Furthermore, distinctive differences in the microbiomes and metagenomes of high- and low-emitting cattle were identified.

## Results

### Methane emissions

Thirty-six Aberdeen Angus and 36 Limousin cross bred steers received one of two diets, one mainly concentrate-based and the other a forage-concentrate-based diet, with forage:concentrate ratios (DM basis) of 8:92 and 48:52, respectively. All cattle came from the same breeding herd population and were kept in the identical farm environment throughout their lifetimes. Methane emissions were measured in respiration chambers and samples of ruminal digesta were obtained at slaughter 1–2 weeks afterwards [30]. Four pairs of digesta samples for each breed/diet combination were selected, based on being which were obtained from animals showing the highest and lowest CH_4_ emissions when expressed in terms of DM intake (Table 1). On average, the high emitters produced 1.88× more CH_4_ than low emitters. Average feed intake was similar between high and low emitters, at 10.59, SE 1.03, kg DM intake/d and 10.68, SE 1.03, kg DM intake/d, respectively.

**Table 1:** Estimation of abundance of 18S and 16S rRNA genes by qPCR in four pairs of steers with extreme methane emissions

	AA/Conc		L/Conc		AA/Med		L/Med		Mean		P value	Ratio (H/L)
	Low	High	Low	High	Low	High	Low	High	Low	High		
CH_4_ (g/kg DM intake)	7.63	18.14	9.29	20.13	17.41	32.42	19.37	30.37	13.43	25.26		1.88
qPCR of 16S and 18S rRNA genes (copies/ng DNA)												
Protozoa	9395	8315	464	18865	500	24480	20940	1768	7825	13357	0.613	1.71
Bacteria	923503	1026155	964081	572312	891432	575574	563798	595201	835704	692311	0.329	0.83
Archaea	23269	72076	21459	40242	19662	54880	37006	75912	25349	60777	0.011	2.40
Cluster IV	110080	278085	114411	76922	135520	221092	175738	236213	133937	203078	0.201	1.52
Cluster XIVa	193249	119584	173592	99130	181399	89060	97723	155352	161491	115782	0.279	0.72
Bacteroidetes	337544	276658	215824	299295	342174	134542	274886	122765	292607	208315	0.277	0.71
Proteobacteria	3341	^a^	9783	1446	21270	2085	4350	2489	11801^a^	2007^a^	0.192	0.17

Comparison of methane emissions (g/kg DM intake) and the abundance of various bacterial taxa as determined by qPCR for 4 pairs of steers matched for breed and diet. *AA* Aberdeen Angus, *L* Limousin. Conc, high concentrate diet; Med, mixed forage: concentrate diet. *DMI* dry matter intake

^a^Sample produced an abnormal melt curve. The mean was based on the three other pairs

### Microbial community analysis

qPCR of specific regions of 16S and 18S rRNA genes enabled a characterisation of the broad features of the microbiome (Table 1). Although protozoa and *Clostridium* Cluster IV bacteria were on average 1.71× and 1.52× more abundant, respectively, in high emitters and *Clostridium* Cluster IV and Bacteroidetes were 0.72× and 0.71× as abundant, the only difference that was statistically significant (*P* = 0.011) was a 2.40× higher archaeal abundance in high emitters. The proportion of the abundance of archaeal to bacterial 16S rRNA genes (the A:B ratio^(30)^) was on average 3 % in low emitters and 9 % in the high emitters. Bacteroidetes comprised 33.4 % of the bacteria, while *Clostridium* Cluster IV and XIVa were 23.4 and 18.3 %, respectively. Proteobacteria appeared to be less abundant in high emitters, but DNA from one sample proved impossible to amplify to produce an acceptable melt curve.

Genomic DNA extracted from the same samples was also submitted for deep sequencing using the Illumina HiSeq platform. Reads mapping to 16S rRNA gene sequences were assigned to taxonomic groups at kingdom, phylum and genus levels (Table 2). Archaeal abundance was, as with qPCR above, calculated relative to bacterial abundance. As with qPCR, the archaea were more abundant (2.49×; *P* = 0.046) in the high emitters, reflected in similar differences in Euryarchaeota at the phylum level and *Methanobrevibacter* at genus level. *Methanobrevibacter* abundance varied from 1.9 to 11.0 % compared to total bacterial counts and was 2.54× more abundant (*P* = 0.017) in high emitters. *Methanosphaera* were much less abundant (0.01 to 0.13 %), while *Methanobacterium* (0 to 0.03 %) were not identified or of very low abundance still (Table 2). *Methanosphaera* were 2.44× more abundant (*P* = 0.014) in high emitters. *Thermoplasmata-*related archaea were less abundant than other archaeal genera and their abundance was greater in the high emitter in only three of the four pairs.

**Table 2:** Estimation of abundance of 16S rRNA genes in ruminal metagenomic sequences from four pairs of steers with extreme methane emissions (% of mapped reads)

	AA/Conc		L/Conc		AA/Med		L/Med		Mean			
	Low	High	Low	High	Low	High	Low	High	Low	High	*P* value	Ratio (H/L)
Domain												
Bacteria	98.84	97.72	99.24	95.08	98.82	96.60	97.07	95.60	98.49	96.25	0.0455	0.98
Archaea	1.16	2.28	0.76	4.92	1.18	3.40	2.93	4.40	1.51	3.75	0.0455	2.49
Bacteria												
Phylum/Class												
Firmicutes	45.25	43.11	48.81	40.47	43.93	46.31	39.50	52.74	44.37	45.66	0.9866	1.03
Bacteroidetes	36.21	40.06	25.70	45.33	34.53	36.47	49.98	31.64	36.61	38.38	0.3975	1.05
Proteobacteria	11.93	3.71	17.15	4.44	13.17	1.92	2.56	0.83	11.20	2.73	0.0015	0.24
Actinobacteria	1.88	1.38	1.90	1.10	1.01	1.02	0.57	1.17	1.34	1.17	0.1309	0.87
Cyanobacteria	1.69	1.41	2.11	0.78	0.07	0.42	0.10	0.17	0.99	0.69	0.2421	0.70
Tenericutes	0.73	2.27	1.79	1.27	1.65	2.48	0.86	1.87	1.25	1.97	0.2425	1.57
Spirochaetes	0.46	3.65	1.27	0.54	1.39	0.89	0.55	1.15	0.92	1.56	0.5873	1.70
Lentisphaerae	0.38	0.38	0.17	0.05	0.01	0.84	0.27	0.81	0.21	0.52	0.2491	2.50
TM7	0.12	0.31	0.06	0.05	0.50	0.38	0.60	0.49	0.32	0.31	0.5073	0.96
Synergistetes	0.10	0.15	0.07	0.18	0.02	0.08	0.07	0.12	0.07	0.13	0.0215	1.95
Verrucomicrobia	0.03	0.80	0.03	0.27	0.59	2.69	1.16	2.48	0.45	1.56	0.0695	3.44
Fibrobacteres	0.02	0.09	0.13	0.04	1.65	1.92	0.05	0.85	0.46	0.73	0.2181	1.57
Genus												
*Prevotella*	54.11	38.09	45.60	57.30	50.04	39.96	56.00	30.41	51.44	41.44	0.3067	0.81
*Butyrivibrio*	10.74	6.20	5.93	7.12	1.68	7.79	6.56	17.44	6.23	9.64	0.3757	1.55
*Succiniclasticum*	5.59	6.26	14.34	11.05	6.83	4.21	6.34	4.84	8.28	6.59	0.1097	0.80
*Bulleidia*	5.51	1.14	3.66	3.05	0.56	0.29	0.21	0.56	2.49	1.26	0.1487	0.51
*Sharpea*	3.69	0.13	0.50	0.53	1.93	0.31	0.05	0.33	1.54	0.33	0.1228	0.21
*Ruminococcus*	2.94	10.29	3.25	1.42	3.92	18.43	11.30	13.30	5.35	10.86	0.1288	2.03
YRC22	1.92	7.53	6.80	0.60	0.35	1.85	1.98	1.58	2.76	2.89	0.9796	1.05
*Acidaminococcus*	1.65	0.21	2.47	0.47	0.05	0.01	0.01	0.00	1.04	0.17	0.0711	0.17
*Coprococcus*	1.32	2.56	2.36	1.21	2.30	1.23	1.06	1.14	1.76	1.53	0.5241	0.87
*Roseburia*	1.10	0.03	0.46	0.00	0.29	0.02	0.01	0.02	0.47	0.02	0.0484	0.04
*Treponema*	0.87	7.80	3.07	0.85	2.97	1.45	0.81	2.47	1.93	3.14	0.6840	1.63
*Megasphaera*	0.82	0.05	0.46	0.06	0.70	0.05	0.01	0.00	0.50	0.04	0.0056	0.08
*Shuttleworthia*	0.78	0.49	2.81	0.47	0.15	0.04	0.14	0.10	0.97	0.27	0.1718	0.28
*Pseudoramibacter_Eubacterium*	0.61	0.03	0.59	0.22	0.29	0.04	0.00	0.04	0.37	0.08	0.0118	0.22
*Mogibacterium*	0.51	1.32	0.33	1.15	0.56	1.84	2.12	3.34	0.88	1.92	0.0407	2.17
CF231	0.43	1.11	0.04	0.19	0.88	1.74	1.28	1.00	0.66	1.01	0.0492	1.53
*Bifidobacterium*	0.39	0.04	0.01	0.03	0.82	0.01	0.08	0.13	0.32	0.05	0.1569	0.17
*Mitsuokella*	0.38	0.20	0.12	0.04	0.04	0.00	0.01	0.00	0.14	0.06	0.0375	0.43
*Lactobacillus*	0.35	0.40	0.77	0.10	0.29	0.18	0.08	0.17	0.37	0.21	0.2347	0.57
*Succinivibrio*	0.32	3.97	0.32	0.12	0.26	0.03	0.07	0.02	0.24	1.03	0.4932	4.32
*Anaerostipes*	0.31	0.04	0.03	0.00	0.29	0.16	0.02	0.17	0.16	0.09	0.1915	0.56
*Blautia*	0.29	0.39	0.17	0.17	0.87	0.38	0.22	0.56	0.39	0.37	0.7396	0.97
*[Eubacterium]*	0.26	0.02	0.03	0.15	5.37	0.00	0.04	0.00	1.43	0.04	0.2619	0.03
*Oscillospira*	0.24	0.50	0.25	0.26	0.25	0.36	0.23	0.41	0.24	0.38	0.0816	1.58
RFN20	0.20	0.15	0.10	0.17	1.15	0.93	0.14	0.51	0.40	0.44	0.7813	1.11
*Bacteroides*	0.20	0.17	0.03	0.58	0.43	0.28	0.27	0.22	0.23	0.31	0.6107	1.34
*Dialister*	0.17	0.03	0.75	0.00	0.40	0.00	0.00	0.00	0.33	0.01	0.0491	0.02
*Clostridium*	0.15	0.36	0.06	0.05	2.04	0.63	0.31	0.78	0.64	0.45	0.5288	0.70
*Desulfovibrio*	0.13	0.29	0.21	0.34	0.11	0.27	0.16	0.34	0.15	0.31	0.0013	2.04
*Pyramidobacter*	0.13	0.24	0.12	0.24	0.05	0.12	0.09	0.22	0.10	0.20	0.0045	2.10
*Moryella*	0.12	0.70	0.09	0.48	0.32	0.51	0.49	0.88	0.25	0.64	0.0212	2.54
*Selenomonas*	0.12	1.29	0.33	0.98	0.55	0.68	1.13	0.34	0.53	0.82	0.2378	1.55
*Catenibacterium*	0.09	0.00	0.00	0.01	0.07	0.02	0.00	0.01	0.04	0.01	0.1282	0.24
*Dorea*	0.09	0.02	0.14	0.02	0.04	0.01	0.01	0.04	0.07	0.02	0.0671	0.31
Archaea												
Euryarchaeota	1.18	2.31	0.77	4.98	1.20	3.42	2.98	4.47	1.53	3.79	0.0266	2.48
Crenarchaeota	0.01	0.01	0.00	0.04	0.00	0.04	0.02	0.01	0.01	0.02	0.2152	3.00
Methanobacteria	1.13	2.20	0.73	4.83	1.15	3.25	2.84	4.27				
Methanococci	0	0	0	0	0	0	0	0				
Methanobacterium	0	0	0	0	0	0	0	0				
*Methanobrevibacter*	2.27	4.93	1.88	8.40	2.73	7.49	5.62	11.00	3.13	7.95	0.0169	2.54
*Methanosphaera*	0.01	0.04	0.01	0.05	0.06	0.08	0.07	0.13	0.04	0.07	0.0141	2.44
Thermoplasmata	0.02	0.07	0.03	0.03	0.10	0.06	0.12					

Among the bacteria, Firmicutes and Bacteroidetes were not different, in contrast to Proteobacteria, which were 0.24× less abundant in the high emitters (*P* = 0.002). Analysis of the Proteobacteria was only resolved to family level (Additional file 1: Table S1) due to short reads. Succinivibrionaceae were the most abundant in all samples, averaging 88 % of Proteobacteria reads, average 97 % in low emitters and 79 % in high emitters. Synergistetes were 1.95× more abundant in high emitters (*P* = 0.022). At genus level, *Desulfovibrio* was the genus whose abundance differed most significantly (*P =* 0.001), being twice as numerous in the high emitters. Others that were different included *Megasphaera*, which was only 0.08× as abundant in high emitters (*P =* 0.006). *Mitsuokella* and *Dialister* were lower and *Mogibacterium* and *Pyramidobacter* higher (*P* < 0.05) in high emitters compared to low emitters.

The richness and relative abundance of the bacteria and archaeal genera did not change significantly between low and high emitting animals. Mean Shannon index was 3.17 for high and 2.98 for low emitting animals respectively. Mean Chao1 index estimated 170 genera for high and 172 low emitting animals respectively (Additional file 2: Table S2).

### Gene abundance analysis

Reads were mapped to gene sequences in the KEGG [31] database and analysed (a) in a directed manner towards genes involved directly in methanogenesis, (b) in a directed manner towards genes involved in alternatives to methanogenesis, (c) in a directed manner towards genes involved in methane oxidation, and (d) in a global manner to compare the abundances of all annotated genes in the metagenome. The whole KEGG dataset can be seen in Additional file 3: Table S3. Statistical *P* values in Additional file 3: Table S3 are uncorrected for multiple comparisons. When the data are discussed within their biological context, uncorrected *P* values are quoted below. When multiple testing was accounted for in a partial least squares analysis, few significant differences due to KEGG genes were found (Table 3), but many of these were genes associated with methanogenesis or archaea, below. The genes identified in Table 3 to be important explained the variation in methane emissions by 88 %.

**Table 3:** Partial least squares estimates of KEGG genes and diet effects and the variation in methane emissions. Partial least squares estimates of KEGG genes and diet effects in an analysis where the partial least squares factors^a^ explaining 87.6 % of the variation of model effects and 87.9 % of the variation in methane emissions

KEGG ID/Diet	Description	^b^Estimate	^c^VIP
K06001	Tryptophan synthase beta chain [EC:4.2.1.20]	0.13310	1.233
Diet conc	Concentrate based diet	−0.14695	1.204
Diet mixed	Mixed forage-concentrate diet	0.14695	1.204
K02118	**V/A-type H** ^**+**^ **-transporting ATPase subunit B [EC:3.6.3.14]**	0.07984	1.151
K02117	**V/A-type H** ^**+**^ **-transporting ATPase subunit A [EC:3.6.3.14]**	0.08564	1.133
K00638	Chloramphenicol O-acetyltransferase [EC:2.3.1.28]	0.06182	1.082
K00200	**Formylmethanofuran dehydrogenase subunit A [EC:1.2.99.5]**	0.06042	1.070
K03531	Fell division protein FtsZ	0.07526	1.065
K00201	**Formylmethanofuran dehydrogenase subunit B [EC:1.2.99.5]**	0.04859	1.049
K00399	**Methyl-coenzyme M reductase alpha subunit [EC:2.8.4.1]**	0.03843	1.021
K00123	**Formate dehydrogenase, alpha subunit [EC:1.2.1.2]**	0.03417	1.013
K03388	**Heterodisulfide reductase subunit A [EC:1.8.98.1]**	0.02734	0.997
K14126	**F**_**420**_ **-non-reducing hydrogenase subunit A [EC:1.12.99.-]**	0.00384	0.933
K01079	Phosphoserine phosphatase [EC:3.1.3.3]	0.03424	0.932
K00401	**Methyl-coenzyme M reductase beta subunit [EC:2.8.4.1]**	0.00484	0.909
K00527	Ribonucleoside-triphosphate reductase [EC:1.17.4.2]	0.03449	0.898
K02337	DNA polymerase III subunit alpha [EC:2.7.7.7]	−0.05338	0.886
K02837	Peptide chain release factor RF-3	0.00883	0.852
K01893	Asparaginyl-tRNA synthetase [EC:6.1.1.22]	−0.06673	0.812
K00925	Acetate kinase [EC:2.7.2.1]	−0.00903	0.812
K00656	Formate C-acetyltransferase [EC:2.3.1.54]	−0.00834	0.787
K02948	Small subunit ribosomal protein S11	−0.00400	0.734

Genes in bold type are archaeal genes associated with methane production

^a^Two factors were significant in the partial least squares analysis

^b^Estimates based on predictors and responses to be centred and scaled to have mean 0 and standard deviation 1

^c^Variable Importance for Projection (VIP) statistic of Wold [67], which summarizes the contribution of a variable marker to the model

**Fig. 1:**
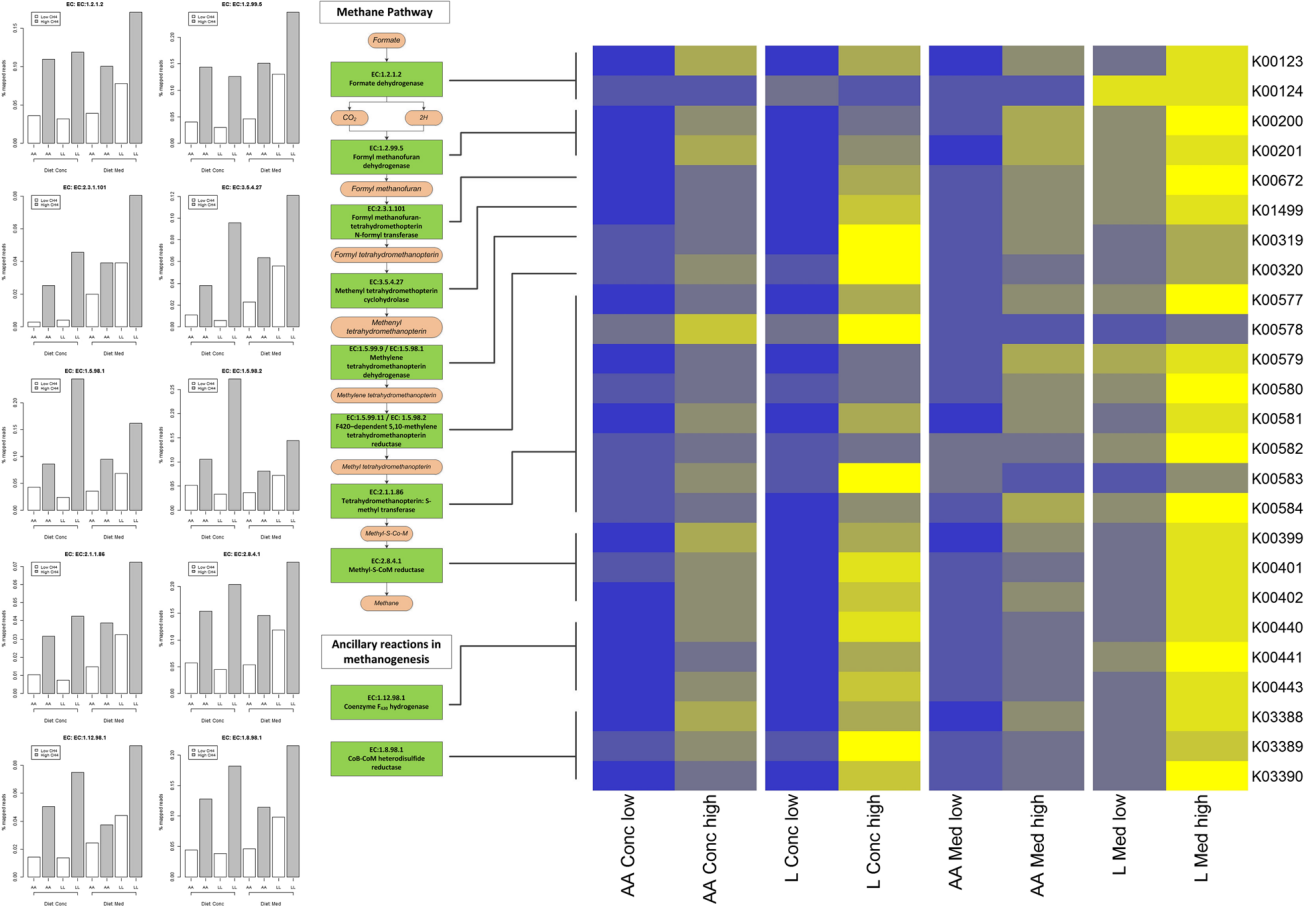
The metagenomic abundance of key elements of the methane production pathway Centre pane: the methane production pathway, plus ancillary reactions, showing enzyme classification (EC) numbers. Left pane: the abundance of each of the relevant EC numbers in our data set. Bar charts show percentage of reads mapped to each enzyme in the 8 samples, 4 pairs of cattle matched for breed (AA = Aberdeen Angus; L = Limousin cross) and diet (High or Medium concentrate). Grey bars are cattle selected for high methane production, white bars are cattle selected for low methane production. For every single enzyme, within each pair, the abundance of the enzyme is higher in high methane producers than in low methane producers. Right pane: heatmap of KEGG orthologues for the EC numbers involved in methane production (lines connect the heatmap to the methane production pathway indicating which K0 numbers represent the given enzymes). The heatmap shows pairs of cattle matched for breed (AA = Aberdeen Angus; L = Limousin cross) and diet (High or Medium concentrate). Blue represents low abundance and yellow represents high abundance. Within each pair, the high methane emitters are to the right and the low methane emitters to the left. A clear pattern emerges – within each pair, these K0 numbers are more abundant (*yellow*) in the high methane emitters than in the low methane emitters

(a) Genes directly involved in methanogenesis. Genes encoding enzymes that are directly involved in methanogenesis [24] were analysed for their abundance in high- and low-emitting cattle (Fig. 1). With the exception of the very low-abundance formate dehydrogenase β subunit (Additional file 3: Table S3), the relative abundance of all genes directly involved in methanogenesis was similar (mean 2.82, SD 0.27, times greater in high emitting animals). The relative abundances of the genes encoding interacting enzymes, coenzyme F_420_ hydrogenase (EC:1.12.98.1) and heterodisulfide reductase (EC:1.8.98.1), were similar to each other and to the genes of the main pathway (mean 2.77, SD 0.20, times greater in high emitting animals). Phosphoserine phosphatase (EC:3.1.3.3) was similarly more abundant in high emitters (2.72×, *P* = 0.040). Most uncorrected *P* values associated with the high-low differences were <0.05, many being much lower (Additional file 4: Table S4).

(b) Genes directly involved in acetogenesis. Acetogenesis by the Ljungdahl-Wood reductive acetogenesis pathway can be divided into three parts [32]. The first part involves the synthesis of 5-methyl-tetrahydrofolate. The first enzyme is an NADP-dependent formate dehydrogenase (EC:1.2.1.43). Only two of the 5.3 million gene assignations mapped to this gene (Additional file 3: Table S3). Enzymes that involve the metabolism of formate *via* tetrahydrofolate (THF) intermediates to 5-methyl-THF, including 10-formyl-THF synthetase (EC:6.3.4.3), 10-formyl-THF deformylase (EC:3.5.1.10), 10-formyl-THF cyclohydrolase (EC:3.5.4.9), 5-formyltetrahydrofolate cyclo-ligase (EC:6.3.3.2), 5,10-methylene-THF dehydrogenase (EC:1.5.1.5) and 5,10-methylene tetrahydrofolate reductase (EC:1.5.1.20 (NADPH)) were present at higher abundance, but none differed significantly between the high and low emitters (Additional file 4: Table S4). Reduction of CO_2_ to CO initiates the capture of CO_2_ [32]; carbon monoxide dehydrogenase iron sulfur subunit (K00196) was 2.22× more abundant in high emitters (*P* = 0.017) and CO dehydrogenase maturation factor (K07321) 2.57× more abundant, though not significantly so (*P* = 0.180). The carbon monoxide dehydrogenase/acetyl-CoA synthase reaction catalysed by EC:2.3.1.69 [33] is the only enzyme that is thought to be unique to reductive acetogenesis [34]; none of the reads was assigned to this gene, despite its having been found in several ruminal bacteria [34].

(c) Genes associated with methanotrophy, methane monooxygenase (EC:1.14.18.3) and methanol dehydrogenase (EC:1.1.2.7), were not identified in the dataset.

(d) The KEGG data were assembled into genes with an abundance of >0.01 % and that differed between high- and low-emitters by uncorrected *P* < 0.05 (Additional file 4: Table S4). The genes directly involved in methanogenesis ((a) above) feature significantly among the increased gene abundances in this Table. Among the 125 genes thus identified, several hypothetical proteins appear. Among the genes whose abundance was higher in high emitters (and not appearing under (a)) were genes associated with methane, hydrogen or archaeal energy metabolism: a H_2_-dependent 5,10-methenyltetrahydromethanopterin hydrogenase (EC:1.12.98.2) (4.31×); cobalt/nickel transport system permease protein (K02007; 2.88×) would help provide Ni to the Ni-dependent methyl reductases, hydrogenases and coenzyme F_430_ involved in methanogenesis; hydrogenase nickel incorporation protein HypB was 2.48× more abundant in high emitters (*P* = 0.004). Genes encoding subunits of V-type H^+^-transporting ATPase (EC:3.6.3.14) were 2.18–2.87× more abundant in high emitters. Subunits of pyruvate ferredoxin oxidoreductase (EC:1.2.7.1) were on average 2.96× more abundant in high emitters (Additional file 5: Table S5).

Among other genes associated with hydrogenase activity, F_420_-non-reducing hydrogenase (EC:1.12.99.-) subunits A, D and G were 2.64×, 3.58× and 3.01× more abundant in high emitters. Likewise, hydrogenase expression/formation protein (K07388) was 2.52× more abundant, along with hydrogenase maturation protein HypF 2.54×; hydrogenase expression/formation protein HypC 5.17×, HypD 2.66× and HypE 2.94×. Energy-converting hydrogenase A subunits B 4.15×, C 3.75×, E 2.53×, G 3.00×, H 2.52×; J 2.65×, M 2.32×, N 3.02×, O 2.11×, P 2.39×, Q 3.31× and R 3.67× and were more abundant in high emitters as were energy-converting hydrogenase B subunits A 4.12×, F 3.33×, H 3.60×, K 2.67×, L 2.85×, M 2.53× and N 2.59×.

Two genes of the pentose phosphate pathway tended to be less abundant, glucose-6-phosphate isomerase (EC:5.3.1.9, 0.615×, *P* = 0.054) and transketolase (EC:2.2.1.1, 0.692×, *P* = 0.104). Transaldolase (EC:2.2.1.2, 0.389×, *P* = 0.279) was numerically less abundant, but other enzymes of this pathway were either not identified or not different (EC:3.1.1.17, 1.1.1.49, 3.1.1.17, .1.1.1,44, 5.1.3.1; Additional file 4: Table S4).

Other genes that were of higher abundance in high emitters included transcription initiation factor TFIIB (K03124, 2.63×, *P =* 0.009), which is archaeal, and transcription initiation factor TFIID TATA-box-binding protein (K03120; 4.31×, *P =* 0.027), which is present in all domains. The enzymes NADH dehydrogenase (EC:1.6.99.3, 2.60×, *P =* 0.018) and (R)-2-hydroxyacid dehydrogenase (EC:1.1.1.272, 4.09×, *P =* 0.024) were also more abundant in high emitters.

Genes that were less abundant in high emitters included electron transport complex proteins RnfC (K03615, 0.357×, *P* = 0.010) and RnfD (K03614, 0.550×, *P* = 0.027), and F-type H^+^-transporting ATPase subunit a (EC:3.6.3.14, 0.620×, *P* = 0.028) (Additional file 4: Table S4). Acetate kinase was lower (EC:2.7.2.1, 0.559×, *P* = 0.024) and there were trends for the related enzymes, phosphate acetyl transferase (EC:2.7.2.1, acetyl-CoA:phosphate acetyltransferase, 0.596×, *P* = 0.066) and pyruvate kinase (EC:2.7.1.40, 0.366×, *P* = 0.074) also to be lower (Additional file 4: Table S4). Others included saccharopine dehydrogenase (NAD^+^, L-lysine forming) (EC:1.5.1.7, 0.549×, *P* = 0.039) and replicative DNA helicase (EC:3.6.4.12, 0.555×, *P* = 0.015].

The positions of several genes on the KEGG metabolic pathways involved in and related to methanogenesis are highlighted in Additional file 6: Figure S1.

### De novo assembly and gene prediction

Metagenomic data were assembled de novo, and predicted genes and proteins were annotated using Pfam [35]. Across all eight samples, there were 1,500,390 predicted proteins, of which a Pfam domain could be assigned to 729,736 (48.6 %). Of those, 97,214 were DUF domains – “domain of unknown function”. Therefore a domain could not be assigned to over half of the predicted proteins, and a putative function to almost 58 %.

Of the 20 KEGG orthologues in Table 3 significantly associated with methane emissions, 2774 putative full length members were discovered in our data set (Table 4). These were searched against NR using BLAST [36]. Of the 2774 predicted proteins, only 16 (0.6 %) gave an exact match in NR, and only 34 (1.2 %) showed 100 % amino acid conservation. Furthermore, only 407 (14.7 %) showed 90 % identity with a protein in NR and only 990 (35.7 %) showed 90 % amino acid conservation.

**Table 4:** Counts of predicted rumen metagenomic proteins and their KO group. Summary of 2774 predicted proteins from the 8 metagenomic rumen samples. KEGG ID is the predicted K0 group. Columns show the number of full length candidates within each K0 group, the number which have 100 % identical matches in NR, the number showing 100 % conservation with a protein in NR, the number with a 90 % identical match, and the number showing at least 90 % conservation with a protein in NR

KEGG ID	Description	# full length candidates	# 100 % identical in NR	# 100 % conserved in NR	# 90 % identical NR	# 90 % conserved in NR
K06001	Tryptophan synthase beta chain [EC:4.2.1.20]	194	1	1	32	78
K02118	V/A-type H^+^-transporting ATPase subunit B [EC:3.6.3.14]	337	2	4	65	169
K02117	V/A-type H^+^-transporting ATPase subunit A [EC:3.6.3.14]	105				21
K00638	Chloramphenicol O-acetyltransferase [EC:2.3.1.28]	76	3	3	7	25
K00200	Formylmethanofuran dehydrogenase subunit A [EC:1.2.99.5]	57			2	8
K03531	Cell division protein FtsZ	167			3	4
K00201	Formylmethanofuran dehydrogenase subunit B [EC:1.2.99.5]	20			3	7
K00399	Methyl-coenzyme M reductase alpha subunit [EC:2.8.4.1]	1			1	1
K00123	Formate dehydrogenase, alpha subunit [EC:1.2.1.2]	3				1
K03388	Heterodisulfide reductase subunit A [EC:1.8.98.1]	352	1	1	26	75
K14126	F_420_-non-reducing hydrogenase subunit A [EC:1.12.99.-]	6			1	4
K01079	Phosphoserine phosphatase [EC:3.1.3.3]	0	0	0	0	0
K00401	Methyl-coenzyme M reductase beta subunit [EC:2.8.4.1]	2				
K00527	Ribonucleoside-triphosphate reductase [EC:1.17.4.2]	120			24	57
K02337	DNA polymerase III subunit alpha [EC:2.7.7.7]	108			5	12
K02837	Peptide chain release factor RF-3	569	2	5	103	271
K01893	Asparaginyl-tRNA synthetase [EC:6.1.1.22]	437		3	54	155
K00925	Acetate kinase [EC:2.7.2.1]	132	1	1	23	38
K00656	Formate C-acetyltransferase [EC:2.3.1.54]	101			18	54
K02948	Small subunit ribosomal protein S11	178	6	16	49	57

Predicted proteins, and their domains, are available as a Meta4 database [37] at http://www.ark-genomics.org/tools/meta4

## Discussion

In the present study, ruminal digesta samples from beef cattle with extreme high and low emissions, identified from an earlier 72-animal study [29, 30], were used to pinpoint key differences in their microbial communities and metagenomes that might help explain the methane phenotype and thereby offer new avenues to explore means of mitigation. Methane emissions correlated with archaeal abundance in the rumen based on 16S rRNA genes and the abundance of other archaeal genes, particularly those involved directly or indirectly with methanogenesis, was greater in high methane emitters. Differences in other members of the rumen microbial community pointed for the first time to a role of Succinovibrionaceae in low methane emissions in ruminants; bacterial gene abundances were consistent with this interpretation. The results of protein mapping analysis from metagenomic sequences highlight that the majority of genes and proteins had no homologues in public databases and that there are many differences between high- and low-emitting beef cattle that could be useful for exploitation.

Previous qPCR analysis of the whole 72-animal experimental group suggested a correlation, albeit rather weak, between archaeal abundance and methane emissions from individual animals [30]. It might be argued that the extreme animals, as investigated here, might give a better impression of what opportunities might be available for modulating methane emissions. The differences in methane emissions, expressed per kg DM intake, between selected low and high emitters were substantial (1.88×), thus the causes of these differences might be more easily identified. *Post-mortem* digesta sampling was used here, following our previous discovery that the abundance of archaea relative to bacteria was similar in live cattle when leaving the respiration chambers and when digesta were sampled at slaughter ca. two weeks later [30].

The rumen microbial community comprises mainly ciliate protozoa, anaerobic bacteria and fungi and archaea. Methanogenesis in the rumen occurs predominantly by the hydrogenotrophic route, i.e. 4H_2_ + CO_2_ = CH_4_ + 2H_2_O [5, 6]. The first three microbial groups provide the H_2_, and the archaea carry out methanogenesis. It therefore seems intuitive that methane emissions should correspond to some extent with archaeal abundance in the rumen, from where 87 % of enteric emissions originate in the digestive tract [38]. Yet, except for our previous studies [30], proof of such a correlation has proved elusive [6, 24, 26–28, 39]. The higher proportion of archaea in high emitters in the present study was similar whether calculated from qPCR or deep sequencing reads of 16S rRNA genes. The large difference between high and low emitters may explain why differences in gene abundance may become more evident. Shi et al. [24] found differences in the ruminal transcriptome but not the metagenome that correlated with sheep emitting different amounts of methane. We would submit that there are strong theoretical reasons why methane emissions should be proportional to the abundance of archaea present in the rumen of individual animals rather than transcript abundances. The biomass yield of the archaea must normally be directly proportional to the methane produced, since, with minor possible exceptions such as alcohol utilization [4], methanogenesis is the only mechanism of ATP synthesis available to the archaea. Furthermore, the cytochrome-containing genera [40] have not been reported in the rumen [41], so the molar growth yields of the different genera that are found in the rumen are likely to be similar. Uncoupling between methanogenesis and ATP synthesis [42, 43] could explain a lack of correspondence between archaeal abundance and methane emissions. Such uncoupling occurs at high H_2_ partial pressures in some archaea [44], but is not known in ruminal archaea as far as we are aware. Furthermore, the partial pressure of H_2_ in the rumen is always low [45]. Several different archaeal genera have been found in the rumen in different species of ruminant in different geographical locations [41]. As found here by examining 16S rRNA reads from the metagenomes, *Methanobrevibacter* usually predominates [41, 46, 47]. The abundance of *Methanobrevibacter* varied from 1.9 to 11.0 % compared to bacterial abundance.

A greater abundance of archaea in high emitters would be expected to be a response to rather than the root cause of the difference in emissions, unless major differences in H_2_ emissions were found, which was not the case [29]. The availability of H_2_ limits the rate of ruminal methanogenesis under some circumstances [45]. Thus, methane emissions might be expected to be at least partly dependent on the abundance of H_2_-producing microorganisms. Ciliate protozoa are major producers of H_2_, produced by mitochondrion-like organelles known as hydrogenosomes [48]. They were generally more abundant in high emitters, but the differences were not statistically significant. Kittelmann et al. [49] also did not find links between protozoa and methane. The bacterial Firmicutes phylum, of which the main ruminal members are *Clostridium* Clusters IV and XIV, would contain more H_2_ producers, particularly *Clostridium* Cluster IV in which the main ruminal community members are the highly cellulolytic *Ruminococcus* and several *Eubacterium* spp. [50, 51], than the Bacteroidetes, which generally are net H_2_ utilisers [51]. Trends in this direction occurred, but as observed with the whole animal group [29, 30] no statistically significant differences in their abundance were observed. Stronger associations between methane emissions and abundance of H_2_-producing bacteria in sheep have been reported by Kittelmann et al. [49], who distinguished three ‘ruminotypes’. The high methane emitters generally had a greater community of H_2_-producing bacteria than low emitters.

Highly significant differences were observed in the abundances of some bacterial taxa based on 16S rRNA sequences extracted from the metagenome. Proteobacteria were 4-fold more abundant in low emitters. The predominant Proteobacteria belonged to the family Succinivibrionaceae. This observation has a curious correspondence with the abundance of Succinivibrionaceae in the digestive tract of the Tammar wallaby (9 % of total bacteria) [52], which was considered to be the main reason why the Tammar wallaby produces one quarter of the methane emissions of cattle [52, 53]. Succinivibrionaceae were just as abundant in the low-emission beef cattle investigated here as in the wallaby. These bacteria produce succinate as a main fermentation product, thus trapping metabolic hydrogen rather than releasing it as H_2_. It may be that the main Succinivibrionaceae species are different in the two animal hosts; indeed, Pope et al. [52] did not find any of the major wallaby species of Succinivibrionaceae in cattle. Nonetheless, the finding that Succinivibrionaceae were much more numerous in low-emitting cows is consistent with the wallaby observations and offers a possible strategy for lower methane emissions from ruminant livestock.

Other significant differences in bacteria may not be explained directly in terms of methane emissions, but possible related causes of the differences are of interest. *Desulfovibrio*, like archaea, are H_2_ utilisers, using H_2_ to reduce sulphate to sulphide [54, 55]; their 2-fold higher abundance in high emitters might therefore be linked with a greater availability of H_2_. *Megasphaera* is a genus associated with adaptation of the ruminal community to low pH [56, 57]. Their greater abundance in low emitters could be indicative of a less stable pH in these animals. (Ruminal pH was not measured here, because such measurements made *post mortem* have limited or no value). *Dialister*, from the phylum Firmicutes, family Selenomonadales, which also were much more abundant in low emitters, might fall into a similar category based on their metabolic properties [58]. Ruminal methanogenesis is highly sensitive to low pH [59, 60], thus the cause of the greater abundance of these bacterial genera in low emitters may be consistent with a low-pH inhibition of archaea in these animals. Two unrelated genera that were more abundant in high emitters were *Mogibacterium* and *Pyramidobacter*, both of which are asaccharolytic [61, 62]. Why such a characteristic should enrich for bacteria with this type of metabolism in high methane emitters is unclear. *Quinella* spp., which were found to be more abundant in low-emitting sheep [49], were not resolved in our taxonomic analysis. However, at the family level, the Veillonellaceae, of which *Quinella* is a member, were considerably more numerous in the low-emitting cattle.

Relatively low values of Shannon diversity reflected the low taxonomic richness and dominance of genera such as *Prevotella* and *Butyrivibrio* within the bacteria and *Methanobrevibacter* within the archaea. The lack of any significant difference in diversity statistics between the high and low emitting animals indicated that the generic taxonomic composition of the microbiome was not altered as a result of the breed, diet or methane emission profile. However, it is likely that these measurements of diversity were not sensitive enough to detect differences in microbiome populations associated with methane profiles. Correspondence analysis carried out on larger sample numbers [49] or detailed analyses such as the quantitative PCR carried out here, was required to reveal more subtle changes in the key microbial species involved in methanogenesis.

Bacterial gene abundances that differed in low and high emitters included several involved in acetate formation and pyruvate metabolism. Acetate kinase (EC:2.7.2.1), which catalyses the conversion of acetyl phosphate to acetate with the formation of ATP, was 0.56× as abundant in high emitters. Phosphotransacetylase (EC:2.3.1.8) forms acetyl phosphate from acetyl CoA; its abundance was similarly lower (0.60×) in high emitters. A possible alternative route for acetate formation, acetate thiokinase (EC:6.2.1.1), was unchanged. Pyruvate ferredoxin oxidoreductase (EC:1.2.7.1), which forms acetyl CoA from pyruvate while reducing ferredoxin, showed a higher abundance of its α, β, γ an δ subunits by 2.9, 2.7, 3.2 and 3.2×, respectively, in high emitters. In contrast, pyruvate formate lyase, an alternative route of acetyl CoA formation from pyruvate, had much lower abundance (0.32×) in high emitters. Thus, the ruminal microbiota in high methane emitters metabolised pyruvate differently to low emitters, favouring the pyruvate formate lyase – acetate kinase route. Perhaps significantly, this is the route by which pyruvate is converted to acetate used by Succinivibrionaceae isolate WG-1 from the Tammar wallaby [52]. Two genes of the pentose phosphate pathway characteristic of the wallaby species WG-1 [52] and WG-2 [53] also tended to be more abundant in low emitters.

It was notable that genes which catalyse methane oxidation [63] were absent, suggesting that significant methane oxidation does not occur in beef cattle. Reverse methanogenesis remains a possibility, however [64]. Although some genes associated with reductive acetogenesis, such as carbon monoxide dehydrogenase iron sulfur subunit and CO dehydrogenase maturation factor, were more abundant in high emitters, the only enzyme that is thought to be unique to reductive acetogenesis, carbon monoxide dehydrogenase/acetyl-CoA synthase (EC:2.3.1.69) [33, 34] was not present, despite its having been found in several ruminal bacteria [34].

Multiple comparison analysis was not carried out here, for simple reasons. In Table 2, for example, 16 out of 52 *P* values are significant at *P* < 0.05, which clearly exceeds the false positive rate. As this was an exploratory study, rather than one which aimed to test specific hypotheses, we considered that a multiple testing adjustment of individual *P* values, such as a Bonferroni correction, would severely inflate the false negative rate. We therefore preferred to present unadjusted *P* values, leaving it to the reader to bear in mind that a few (2–3) of them can be expected to be false positives, though most will not.

Among the aims of the type of methane research described here are to find proxies for estimating methane emissions – the respiration chambers used in the present experiments are expensive and laborious, unsuited to large numbers of animals - and to identify targets for interventions to lower methane emissions. Here, comparisons of metagenomic profiles identified differences that characterise high- and low-emitting animals. Table 3 describes the cumulative contribution of a group of genes on methane emissions, considering the correlations among gene effects. Thereby, the effect of each gene is estimated independently from those of all other genes in the model. The order of importance of the genes may reflect therefore more the independent regulatory effects of each gene on methane emissions. Thus, there are several possible strategies for identifying proxies and targets.

Our results demonstrate that the rumen is still a hugely unexplored environment containing many novel enzymes, which could be of significant interest to the agricultural and biotechnology markets. From just 8 samples and a little under 88 Gb of sequencing data, we were able to predict over 1.5 million proteins, the majority of which we were unable to assign function to. Predicting the function of proteins such as these (those with no hits in public databases) will be a major challenges facing biologists in the next decade. Of the proteins we could assign some function to, the vast majority were novel, with over 99 % of them having no exact matches within NR, and the majority showing conservation levels below 90 %. We can predict their function through homology to enzymes and proteins of known function; however, undoubtedly they will differ in their ability to catalyse reactions, and some may be more efficient at their task, which would be of profound interest to a range of researchers and companies in biotechnology. By releasing all 1.5 million proteins, with predicted domains, as a Meta4 database, we allow researcher to explore a dataset of huge importance and impact.

## Conclusion

In conclusion, the results presented here demonstrate that the abundance of archaea and their constituent genes corresponds strongly with methane emissions by the host animal. The gene abundances can now be used individually or collectively as proxies for methane emissions in genetic screening studies. The discovery that pyruvate and acetate metabolism and the numbers of Succinivibrionaceae differ between low and high emitters may bring insight into how metabolic pathways and the microbial community might be manipulated to lower methane emissions and thus lessen the environmental footprint of ruminant livestock production.

## Methods

### Animals, experimental design and diets

This study was conducted at the Beef and Sheep Research Centre of SRUC (6 miles south of Edinburgh, UK) in summer 2011. The experiment was approved by the Animal Experiment Committee of SRUC and was conducted in accordance with the requirements of the UK Animals (Scientific Procedures) Act 1986. Full details of the methodology of animal experimentation have been provided previously [29, 30]. Only an outline is given here.

Thirty-six Aberdeen Angus and 36 Limousin cross bred steers received one of two diets, one mainly concentrate-based and the other a forage-concentrate-based diet, with forage:concentrate ratios (DM basis) of 8:92 and 48:52, respectively. The composition of the diets is given in Additional file 7: Table S6. Feed samples were analysed for DM, ash, CP, ADF and NDF according to standard methods [65]. Gross energy of feeds was performed on dried samples by adiabatic bomb calorimetry. All cattle came from the same breeding herd population and were kept in the identical farm environment throughout their lifetimes.

Eighteen animals of each breed received each diet. Methane emissions were measured individually for 48 h in respiration chambers. Samples of ruminal digesta were recovered at slaughter up to 2 weeks later. The highest and lowest emitter, expressed as g methane per kg DM intake, were identified from each of the breed/diet combinations and the stored DNA was subjected to qPCR of 16S rRNA genes and to deep sequencing.

### qPCR of 16S and 18S rRNA genes

Sample storage and DNA extraction were carried out using methods described and authenticated in previous studies, as described by Rooke et al. [29]. Primers used for amplification of 16S and 18S rRNA genes, amplification protocols, calibration and calculation of gene abundance were also the same as those reported by Rooke et al. [29].

### Deep sequencing and KEGG analysis

Illumina TruSeq libraries were prepared from genomic DNA and sequenced on an Illumina HiSeq 2500 instrument by Edinburgh Genomics. 100-bp paired-end reads were generated, resulting in between 8.6 and 14.5 Gb per sample (between 43.4 and 72.7 million paired reads).

For 16S rRNA gene analysis, the genomic reads were aligned to the GREENGENES database [66] using Novoalign (www.novocraft.com). Parameters were adjusted such that all hits were reported that were equal in quality to the best hit for each read, and allowing up to a 10 % mismatch across the fragment. Taxa were assigned to each read as follows. Where a read hit a single entry in the GREENGENES database, the full taxonomy for that hit was taken. Where a read hit multiple entries in the GREENGENES database, the lowest common taxon was taken. The number of reads that matched at each of Kingdom, Phylum and Genus were counted, normalised to the total number of hits and expressed as a percentage.

The genus abundance table, based on counts, was used to generate diversity statistics for the microbiome in each sample. Shannon index [67] was used as a measure of richness and evenness and Chao1 index [68] to calculate the estimated total number of prokaryotic genera.

For functional analysis, a similar approach was taken. The genomic reads were aligned to the KEGG genes database using the same parameters, read counts for KEGG orthologues summed and normalised to the total number of hits. We first aligned reads directly to KEGG genes allowing for up to a 10 % mismatch. The KEGG Orthologue groups (KO) of all hits that were equal to the best hit were examined. If we were unable to resolve the read to a single KO, the read was ignored; otherwise, the read was assigned to the unique KO.

### Calculations and statistical analysis

Microbial 16S rRNA abundances were compared using paired *t*-tests. Comparisons of gene abundances were carried out as follows. Firstly, a generalized linear model analysis (GLM, Version 9.1 for Windows, SAS Institute Inc., Cary, NC, USA) was performed, fitting diet effects (*P* < 0.05) and one KEGG gene each time. Breed showed non-significant effects (*P* > 0.1) on methane emissions per kg DM intake and therefore was not fitted in the model. Secondly, a partial least squares analysis (PLS, Version 9.1 for Windows, SAS Institute Inc., Cary, NC, USA) was carried out fitting all KEGG genes identified in the GLM analysis to have *P* < 0.1 (including diet effects) in order to account for multiple testing and the correlations among all these model effects. Model effects with a variable importance for projection (VIP) criteria [69] of <0.8 were removed from the model because Wold [69] indicates that those effects contribute little to the prediction. No further PLS analyses were carried out even when a few genes moved below VIP values of 0.8.

### Metagenomic assembly and gene prediction

Each sample was assembled de novo using MetaVelvet [70] and a kmer of 51. From the resulting scaffolds, microbial genes were predicted using Prokka [71], and compared to Pfam [35] using HMMER [72]. All protein predictions and annotations were uploaded to a Meta4 database [37]. We applied a *P*-value cut-off of 0.01 to the resulting domain predictions and counted the number of protein predictions which were assigned domains.

We then searched for members of the 20 KEGG orthologous groups from Table 3 in our dataset. Specifically, for each of the 20 KEGG orthologous groups in Table 3, we searched for predicted proteins that had a similar domain structure and which were greater than or equal to the length of the smallest protein in the group. These were then compared to the NR database using BLAST [36].

## Availability of supporting data

Predicted proteins, and their domains, are available as a Meta4 database [37] at http://www.ark-genomics.org/tools/meta4. Raw data are available in the European Nucleotide Archive under accession PRJEB10338.

## Additional files

Additional file 1: Table S1. Analysis of reads of 16S rRNA assigned to Proteobacteria (DOCX 15 kb) Additional file 2: Table S2. Diversity statistics based on microbial genus abundance in high and low methane emitting cattle. (DOCX 12 kb) Additional file 3: Table S3. KEGG dataset in order of average gene abundance. (XLSX 1400 kb) Additional file 4: Table S4. KEGG dataset in order of the ratio of gene abundance, high:low emitters. (XLSX 443 kb) Additional file 5: Table S5. Gene abundance of genes of pyruvate metabolism in low and high methane steers. (DOCX 14 kb) Additional file 6: Figure S1. KEGG pathways associated with methane metabolism. Highlighted EC gene numbers are those genes that differed significantly between high and low emitting cattle. Red – genes that had higher abundance in high emitters; blue - genes that had lower abundance in high emitters. (DOC 22 kb) Additional file 7: Table S6. Ingredient composition (fresh weight basis; g/kg) of high- concentrate and mixed forage: concentrate diets. (DOCX 11 kb)
