# Supplementary material for: The rumen microbial metagenome associated with high methane production in cattle
Source: BMC Genomics. 2015 Oct 23;16:839. doi: 10.1186/s12864-015-2032-0 (PMC4619255; doi:10.1186/s12864-015-2032-0)
Supplement: Additional file 1: Table S1. — Analysis of reads of 16S rRNA assigned to Proteobacteria (DOCX 15 kb) [file 12864_2015_2032_MOESM1_ESM.docx]

**Supplementary Table S1. Analysis of reads of 16S rRNA assigned to Proteobacteria**

| **Family** | **AA/Conc** | | **L/Conc** | | **AA/Med** | | **L/Med** | | **Mean** | |
| --- | --- | --- | --- | --- | --- | --- | --- | --- | --- | --- |
|  | **Low** | **High** | **Low** | **High** | **Low** | **High** | **Low** | **High** | **Low** | **High** |
| Succinivibrionaceae | 97.04 | 93.98 | 97.01 | 92.02 | 98.76 | 83.83 | 94.26 | 44.44 | 96.77 | 78.57 |
| Enterobacteriaceae | 1.25 | 0.15 | 1.43 | 1.00 | 0.08 | 0.41 | 0.34 | 1.39 | 0.77 | 0.74 |
| Desulfovibrionaceae | 0.65 | 4.55 | 0.53 | 5.19 | 0.37 | 9.43 | 3.86 | 38.43 | 1.35 | 14.40 |
| Alcaligenaceae | 0.37 | 0.27 | 0.34 | 0.17 | 0.26 | 0.21 | 0.17 | 3.70 | 0.28 | 1.09 |
| Campylobacteraceae | 0.24 | 0.19 | 0.20 | 0.45 | 0.03 | 0.00 | 0.13 | 0.46 | 0.15 | 0.28 |
| Comamonadaceae | 0.08 | 0.00 | 0.04 | 0.11 | 0.00 | 0.21 | 0.00 | 0.93 | 0.03 | 0.31 |

All numbers are expressed as a % of the total 16S rRNA genes**.**

AA, Aberdeen Angus; L, Limousin. Conc, high concentrate diet; Med, mixed forage: concentrate diet.
