# Supplementary material for: The rumen microbial metagenome associated with high methane production in cattle
Source: BMC Genomics. 2015 Oct 23;16:839. doi: 10.1186/s12864-015-2032-0 (PMC4619255; doi:10.1186/s12864-015-2032-0)
Supplement: Additional file 2: Table S2. — Diversity statistics based on microbial genus abundance in high and low methane emitting cattle. (DOCX 12 kb) [file 12864_2015_2032_MOESM2_ESM.docx]

Supplementary Table 2. Diversity statistics based on microbial genus abundance in high and low methane emitting cattle.

|  |  | Shannon | Chao1 | Chao1 lower bound | Chao1 upper bound |
| --- | --- | --- | --- | --- | --- |
| AA/Conc | Low | 2.87 | 202 | 148 | 338 |
|  | High | 3.44 | 167 | 140 | 232 |
| L/Conc | Low | 3.11 | 178 | 156 | 228 |
|  | High | 2.55 | 132 | 115 | 173 |
| AA/Med | Low | 3.33 | 155 | 135 | 204 |
|  | High | 3.23 | 237 | 183 | 352 |
| L/Med | Low | 2.62 | 153 | 131 | 207 |
|  | High | 3.48 | 143 | 126 | 183 |
| Mean | Low | 2.98 | 172 | 142 | 244 |
|  | High | 3.17 | 170 | 141 | 235 |
