# Supplementary material for: The rumen microbial metagenome associated with high methane production in cattle
Source: BMC Genomics. 2015 Oct 23;16:839. doi: 10.1186/s12864-015-2032-0 (PMC4619255; doi:10.1186/s12864-015-2032-0)
Supplement: Additional file 5: Table S5. — Gene abundance of genes of pyruvate metabolism in low and high methane steers. (DOCX 14 kb) [file 12864_2015_2032_MOESM5_ESM.docx]

**Supplementary Table S5. Gene abundance of genes of pyruvate metabolism in low and high methane steers**

| **KEGG ID** | **Description** | **Incidence (%)** | | | **Estimate** | **SE** | **P-value** | **R^2^** |
| --- | --- | --- | --- | --- | --- | --- | --- | --- |
|  |  | **Low CH_4_** | **High CH_4_** | **Ratio, H/L** |  |  |  |  |
| K00169 | pyruvate ferredoxin oxidoreductase, alpha subunit [EC:1.2.7.1] | 0.028 | 0.080 | 2.87 | 150.9 | 51.2 | 0.032 | 0.802 |
| K00170 | pyruvate ferredoxin oxidoreductase, beta subunit [EC:1.2.7.1] | 0.020 | 0.053 | 2.65 | 195.6 | 85.1 | 0.070 | 0.737 |
| K00171 | pyruvate ferredoxin oxidoreductase, delta subunit [EC:1.2.7.1] | 0.0036 | 0.0113 | 3.14 | 990.6 | 342.6 | 0.034 | 0.797 |
| K00172 | pyruvate ferredoxin oxidoreductase, gamma subunit [EC:1.2.7.1] | 0.0096 | 0.0304 | 3.17 | 196.4 | 124.5 | 0.176 | 0.639 |
| K00162 | pyruvate dehydrogenase E1 component subunit beta [EC:1.2.4.1] | 0.0003 | 0.0012 | 4.33 | 5820 | 2808 | 0.093 | 0.709 |
| K00163 | pyruvate dehydrogenase E1 component [EC:1.2.4.1] | 0.0007 | 0.0013 | 1.86 | 5087 | 3971 | 0.256 | 0.593 |
| K00123 | formate dehydrogenase, alpha subunit [EC:1.2.1.2] | 0.092 | 0.250 | 2.72 | 58.4 | 15.6 | 0.013 | 0.857 |
| K00125 | formate dehydrogenase, beta subunit [EC:1.2.1.2] | 0.038 | 0.108 | 2.84 | 115.6 | 38.3 | 0.030 | 0.808 |
| K00656 | formate C-acetyltransferase [EC:2.3.1.54] (pyruvate formate lyase) | 0.403 | 0.130 | 0.322 | -22.0 | 9.3 | 0.065 | 0.744 |
| K00625 | phosphate acetyltransferase [EC:2.3.1.8] (phosphotransacetylase) | 0.082 | 0.049 | 0.596 | -244.2 | 67.6 | 0.015 | 0.850 |
| K00925 | acetate kinase [EC:2.7.2.1] | 0.173 | 0.097 | 0.56 | -105.8 | 31.9 | 0.021 | 0.831 |
| K01895 | acetyl-CoA synthetase [EC:6.2.1.1] (acetate thiokinase) | 0.100 | 0.095 | 0.95 | -105.8 | 188.6 | 0.599 | 0.491 |
| K01958 | pyruvate carboxylase [EC:6.4.1.1] | 0.007 | 0.004 | 0.39 | -862.2 | 610.5 | 0.217 | 0.613 |
| K01959 | pyruvate carboxylase subunit A [EC:6.4.1.1] | 0.017 | 0.049 | 2.88 | 247.5 | 84.4 | 0.033 | 0.801 |
| K01960 | pyruvate carboxylase subunit B [EC:6.4.1.1] | 0.164 | 0.174 | 1.06 | 12.2 | 49.0 | 0.813 | 0.466 |
