# Supplementary material for: The rumen microbial metagenome associated with high methane production in cattle
Source: BMC Genomics. 2015 Oct 23;16:839. doi: 10.1186/s12864-015-2032-0 (PMC4619255; doi:10.1186/s12864-015-2032-0)
Supplement: Additional file 6: Figure S1. — KEGG pathways associated with methane metabolism. Highlighted EC gene numbers are those genes that differed significantly between high and low emitting cattle. Red – genes that had higher abundance in high emitters; blue - genes that had lower abundance in high emitters. (DOC 22 kb) [file 12864_2015_2032_MOESM6_ESM.doc]

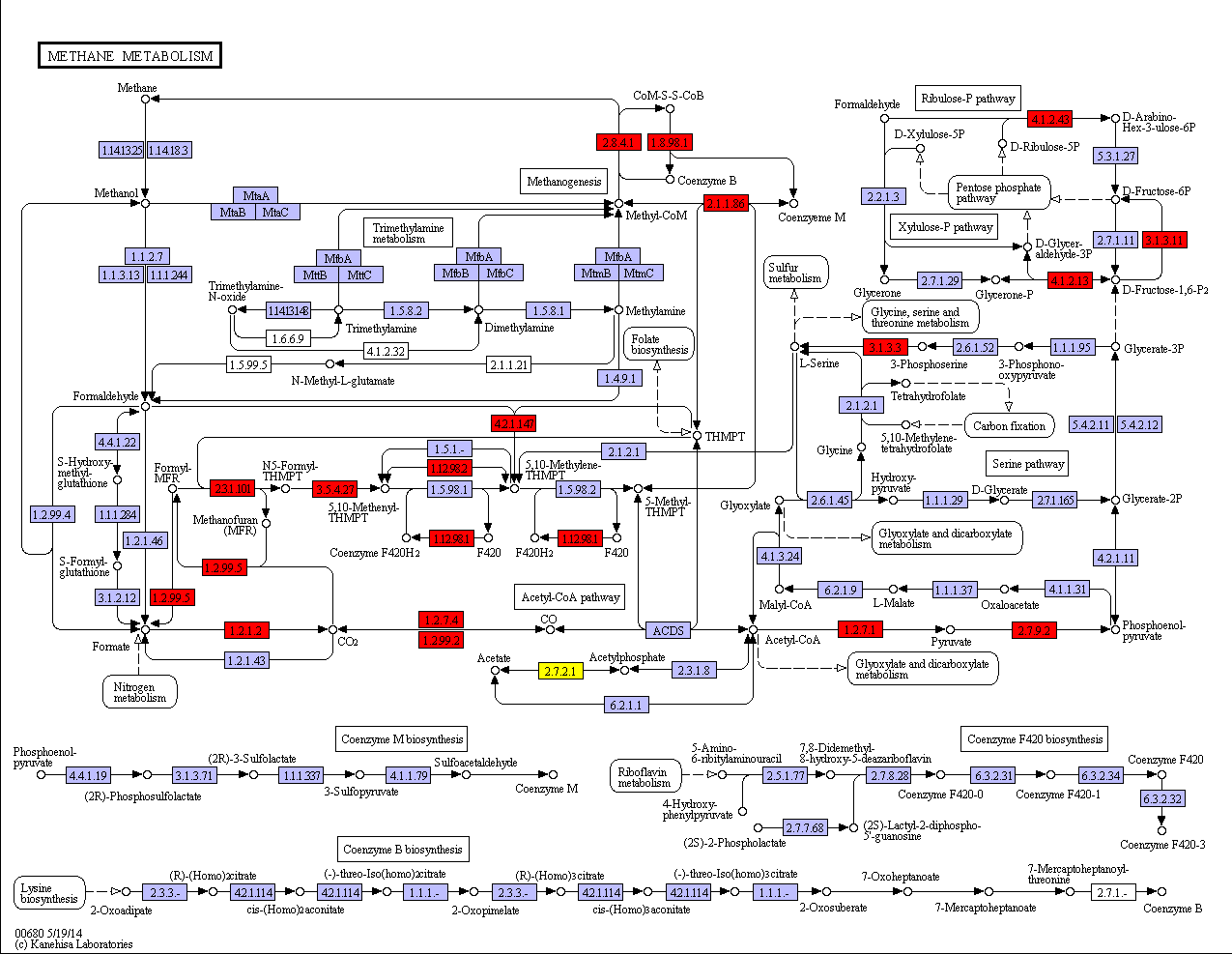


Additional file 6: Figure S1. KEGG pathways associated with methane metabolism. Highlighted EC gene numbers are those genes that differed significantly between high and low emitting cattle. Red – genes that had higher abundance in high emitters; blue - genes that had lower abundance in high emitters.
