# Supplementary material for: The rumen microbial metagenome associated with high methane production in cattle
Source: BMC Genomics. 2015 Oct 23;16:839. doi: 10.1186/s12864-015-2032-0 (PMC4619255; doi:10.1186/s12864-015-2032-0)
Supplement: Additional file 7: Table S6. — Ingredient composition (fresh weight basis; g/kg) of high- concentrate and mixed forage: concentrate diets. (DOCX 11 kb) [file 12864_2015_2032_MOESM7_ESM.docx]

**Supplementary Table S6.** Ingredient composition (fresh weight basis; g/kg) of high- concentrate and mixed forage: concentrate diets

| Ingredient | High concentrate  (Conc) | Mixed forage:concentrate (Med) |
| --- | --- | --- |
| Barley straw | 81 | 0 |
| Grass silage | 0 | 413 |
| Whole crop barley silage | 0 | 340 |
| Barley grain | 688 | 156 |
| Maize distillers dark grains | 200 | 86 |
| Molasses | 20 | 0 |
| Minerals-vitamin supplement* | 10 | 5 |

*Contained (mg/kg): Fe, 6036; Mn, 2200; Zn, 2600; Iodine, 200; Co, 90; Cu, 2500; Se 30; (µg/kg): vitamin E, 2000; vitamin B12, 1000; vitamin A, 151515; vitamin D, 2500
